# Real-world experience with targeted therapy in patients with histiocytic neoplasms in the Netherlands and in Belgium

**DOI:** 10.1016/j.bneo.2024.100023

**Published:** 2024-06-10

**Authors:** Paul G. Kemps, F. J. Sherida H. Woei-A-Jin, Patrick Schöffski, Thomas Tousseyn, Isabelle Vanden Bempt, Friederike A. G. Meyer-Wentrup, Natasja Dors, Natasha K. A. van Eijkelenburg, Marijn A. Scheijde-Vermeulen, Ingrid M. Jazet, Maarten Limper, Margot Jak, Robert M. Verdijk, Marjolein L. Donker, Nick A. de Jonge, Carel J. M. van Noesel, Konnie M. Hebeda, Suzanne van Dorp, Sanne H. Tonino, Jan A. M. van Laar, Cor van den Bos, Astrid G. S. van Halteren, HOVON Histiocytic and Lymphocytic Diseases Working Group, Erik Beckers, Merlijn van den Berg, Cor van den Bos, Godelieve de Bree, Emmeline Buddingh, Kristl Claeys, Paul van Daele, Petra De Haes, Astrid Demandt, Suzanne van Dorp, Liesbeth Hak, Astrid van Halteren, Tim van der Houwen, Margot Jak, Jeroen Kerstens, Arjan Kwakernaak, Jan van Laar, Helen Leavis, Roos Leguit, Arjan van de Loosdrecht, Linde Morsink, Rogier Mous, Max van Noesel, Rimke Oostvogels, Judith Potjewijd, Wouter Plattel, Wilfried Roeloffzen, Abraham Rutgers, Sanne Tonino, Thomas Tousseyn, Rob Verdijk, Joost Vermaat, Sherida Woei-A-Jin

**Affiliations:** 1 Department of Pathology, Leiden University Medical Center, Leiden, The Netherlands; 2 Princess Máxima Center for Pediatric Oncology, Utrecht, The Netherlands; 3 Department of General Medical Oncology, University Hospitals Leuven, Leuven Cancer Institute, Leuven, Belgium; 4 HOVON Histiocytic and Lymphocytic Diseases Working Group, Rotterdam, The Netherlands; 5 Department of Pathology, University Hospitals Leuven, Leuven, Belgium; 6 Translational Cell and Tissue Research Laboratory, Catholic University of Leuven, Leuven, Belgium; 7 Department of Human Genetics, University Hospitals Leuven, Leuven, Belgium; 8 Department of Internal Medicine, Section Endocrinology, Leiden University Medical Center, Leiden, The Netherlands; 9 Department of Rheumatology and Clinical Immunology, University Medical Center Utrecht, Utrecht, The Netherlands; 10 Department of Hematology, University Medical Center Utrecht, Utrecht, The Netherlands; 11 Department of Pathology, Erasmus MC University Medical Center Rotterdam, Rotterdam, The Netherlands; 12 Department of Hematology, Amsterdam University Medical Center, Amsterdam, The Netherlands; 13 Department of Pathology, Amsterdam University Medical Center, Amsterdam, The Netherlands; 14 Department of Pathology, Radboud University Medical Center, Nijmegen, The Netherlands; 15 Department of Hematology, Radboud University Medical Center, Nijmegen, The Netherlands; 16 Department of Internal Medicine, Section Clinical Immunology and Allergology, Erasmus MC University Medical Center Rotterdam, Rotterdam, The Netherlands; 17 Department of Immunology, Erasmus MC University Medical Center Rotterdam, Rotterdam, The Netherlands

## Abstract

Histiocytic disorders are rare hematologic neoplasms characterized by a notable dependence on mitogen-activated protein kinase signaling. Targeted therapy is an emerging treatment option, yet the number of reported patients remains limited. Here, we describe 40 patients with histiocytic neoplasms who were treated with targeted therapy in 7 tertiary referral hospitals from the Netherlands and Belgium. The cohort comprised of 6 (15%) children and 34 (85%) adults with diverse histiocytoses, including Langerhans cell histiocytosis (LCH; n = 12), Erdheim–Chester disease (n = 14), central nervous system xanthogranuloma (n = 2), Rosai–Dorfman disease (n = 3), histiocytic sarcoma (n = 2), ALK–positive histiocytosis (n = 1), and mixed/unclassifiable histiocytosis (n = 6). Five patients were included in a clinical trial; 35 (88%) received BRAF/MEK inhibitors outside of trials. Among these 35 patients with available follow-up data, median time on targeted treatment was 1.9 years (range, 0.04-5.8 years). Complete or partial responses were observed in 25 of 27 (93%) patients treated for multisystemic and/or solid lesions and 2 of 8 (25%) patients treated for neurodegenerative LCH. Responses were generally durable, although 10 patients lost response after dose reduction or therapy interruption. Responses were recaptured in 9 of 10 cases. Two patients developed new or progressive neurodegenerative lesions: 1 during and 1 after vemurafenib therapy. At last follow-up, 8 adults had stopped targeted therapy because of toxicity. This study corroborates the favorable outcomes of BRAF/MEK inhibition in patients with histiocytosis described previously. However, it also highlights limitations and calls for prospective studies.

## Key Points

• Objective responses to BRAF/MEK inhibition were observed across histiocytic neoplasms and regardless of mutational profile.
• Limitations include toxicity, frequent need for prolonged treatment, and rare instances of neurodegeneration despite vemurafenib therapy.

## Visual Abstract

**Figure:**
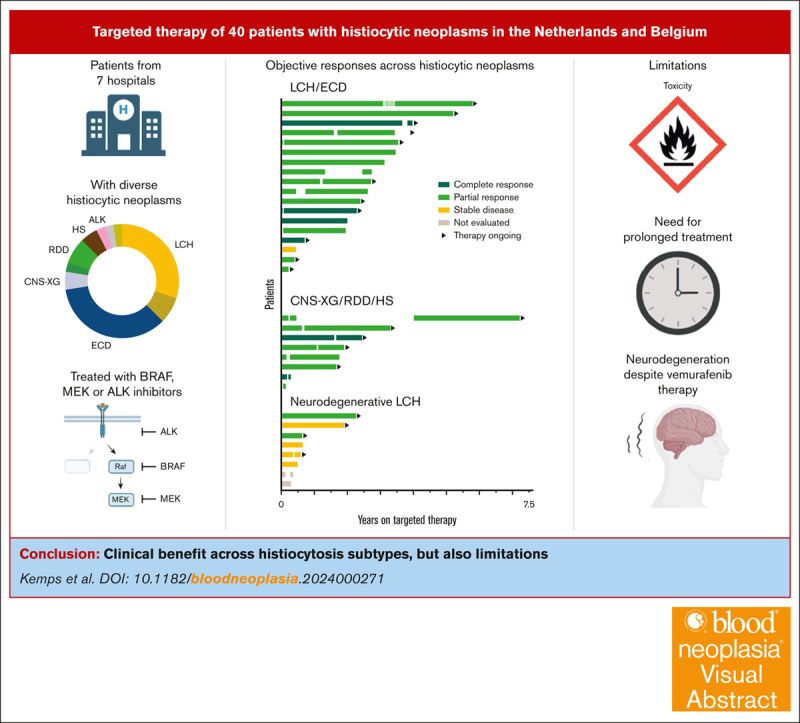

## Introduction

Histiocytic disorders are rare hematologic diseases characterized by the accumulation of monocyte-, macrophage- or dendritic cell–differentiated cells in various tissues.^1^, ^2^, ^3^ Langerhans cell histiocytosis (LCH) is one of the most common histiocytic disorders,^4^ with an incidence rate of 1.6 per million persons per year in a recent study from England.^5^ In 2010, recurrent somatic *BRAF*^*V600E*^ mutations were discovered in LCH.^6^ Subsequently, *BRAF*^*V600E*^ was also detected in other histiocytic disorders,^7^^,^^8^ and alternative somatic alterations in genes of the mitogen-activated protein kinase (MAPK) signaling pathway were identified in histiocytoses without the *BRAF*^*V600E*^ mutation.^9^, ^10^, ^11^, ^12^, ^13^, ^14^, ^15^, ^16^, ^17^ Together, these findings established histiocytic disorders as hematologic neoplasms characterized by a notable dependence on MAPK signaling.^18^

Treatment of histiocytic neoplasms generally depends on the extent of disease and organs involved.^19^, ^20^, ^21^, ^22^ With conventional therapies (eg, immunomodulatory agents; chemotherapy),^19^, ^20^, ^21^, ^22^ a substantial proportion of patients experience disease progression or relapse.^23^, ^24^, ^25^, ^26^, ^27^ Patients with disease involvement of critical organs, such as the central nervous system (CNS) or liver, are particularly difficult to manage. For example, in a large cohort of 30 patients with Erdheim–Chester disease (ECD) and neurologic involvement, conventional therapy led to a radiographic response in less than a third of patients.^26^ Moreover, conventional therapies are rarely effective in patients with neurodegenerative (ND)-LCH or malignant histiocytosis (MH).^28^, ^29^, ^30^, ^31^

ND-LCH is a rare but devastating form of LCH characterized by progressive neurologic deterioration.^29^^,^^32^ It generally presents as a late complication, arising years after initial multisystemic LCH.^33^^,^^34^ Less than 5% of patients with LCH develop the condition.^32^^,^^34^ Clinically, ND-LCH often manifests as a cerebellar syndrome, with variable cognitive impairment.^35^ Radiologically, it is characterized by nontumorous lesions in the cerebellum, basal ganglia, and/or brain stem.^36^ Initially, these lesions were considered to be paraneoplastic or autoimmune phenomena because they lacked CD207^+^ cells typically observed in LCH lesions.^37^^,^^38^ However, recent studies revealed that ND-LCH is likely caused by circulating *BRAF*-mutated myeloid cells infiltrating the brain, where they differentiate into senescent inflammatory macrophages.^39^^,^^40^

MH encompasses the diagnoses of histiocytic sarcoma (HS), Langerhans cell sarcoma, and interdigitating dendritic cell sarcoma, which are immunophenotypic subtypes of histiocytic neoplasms with anaplastic histology.^1^^,^^31^ Prognosis of patients with MH is poor, with a median overall survival of 6 months in a population-based study of 159 patients with HS.^30^ Thus, patients are in dire need of novel treatments. Targeted therapies, which act on specific molecular targets (typically proteins) that play an important role in the development or progression of cancer,^41^ are an emerging treatment option.

First, the B-Raf (BRAF) inhibitor vemurafenib was trialed in several patients with relapsed/refractory *BRAF*^*V600E*^-mutated LCH or ECD.^42^, ^43^, ^44^, ^45^, ^46^, ^47^ These initial studies reported rapid responses. In contrast to patients with *BRAF*^*V600E*^-mutated melanoma, in whom responses to BRAF inhibitors are often short-lived because of tumor escape mechanisms,^48^ patients with histiocytic neoplasms showed durable responses. Given these results, the BRAF inhibitor dabrafenib, the MAPK kinase (MEK) inhibitors cobimetinib and trametinib, and various combinations of BRAF and MEK inhibitors were administered to patients with histiocytosis, with similar results.^18^^,^^49^, ^50^, ^51^, ^52^, ^53^, ^54^, ^55^, ^56^ Yet, the number of reported patients remains limited, particularly of patients with rare histiocytosis subtypes and/or with mutations other than *BRAF*^*V600E*^.

On the basis of 2 phase 2 trials,^18^^,^^44^ the US Food and Drug Administration already granted approval for the use of vemurafenib in the treatment of adults with *BRAF*^*V600*^-mutated ECD,^57^ and for the use of cobimetinib in the treatment of adults with any form of histiocytosis. In Europe, however, no marketing authorization for the treatment of histiocytic neoplasms with BRAF or MEK inhibitors has yet been granted. Consequently, patients must rely on participation in available clinical trials or on their health care providers requesting access to nonreimbursed off-label targeted therapy. Herein, we report our experience in this real-world setting, describing 40 patients with histiocytic neoplasms who were treated with targeted therapy in 7 tertiary referral hospitals from the Netherlands and Belgium.

## Methods

Through a collaborative effort of the Dutch-Belgian Cooperative Trial Group for Hematology Oncology Histiocytic and Lymphocytic Diseases working group, we performed a retrospective, multicenter, observational cohort study of patients with histiocytic neoplasms treated with targeted therapy. Patients were identified by members of the working group, which currently includes histiocytosis experts from all Dutch academic medical centers, the Princess Máxima Center for Pediatric Oncology, and a tertiary referral hospital in Belgium (University Hospitals Leuven) providing care for patients with histiocytosis from across the country and abroad. To capture the variability and complexity of the real-world clinical practice in these centers, patients were included irrespective of age, mutational status, comorbidity/organ dysfunction, targeted agent(s), and treatment history. Targeted therapy was defined as inhibitors of BRAF, MEK or receptor tyrosine kinases known to be involved in the pathogenesis of histiocytoses (anaplastic lymphoma kinase [ALK]/rearranged during transfection[RET]/colony stimulating factor 1 receptor[CSF1R]).^16^^,^^58^ Ethical approval was obtained from the Institutional Review Board of Leiden University Medical Center (nWMO-D4-2022-010) and the Ethics Committee of University Hospitals Leuven (S64542). The study was also approved by the Biobank and Data Access Committee of the Princess Máxima Center for Pediatric Oncology (PMCLAB2022.358). Written informed consent was obtained from patients and/or their legal representatives when required.

Clinical, histopathologic, and molecular information was extracted from the medical records using a standardized case report form. For the few patients enrolled in a clinical trial, outcome data were not collected so as to not interfere with these trials. Responses were classified as complete response (CR), partial response (PR), stable disease (SD), or progressive disease (PD), as previously described.^58^ Responses were based on local clinical interpretations of ultrasound, computed tomography, magnetic resonance imaging (MRI), and/or positron emission tomography (PET) when available. To not overestimate results, the less favorable radiologic response was prioritized if multiple imaging methods that showed discrepant responses (eg, PR by MRI; CR by PET ) were used.^58^ Objective response was defined as PR or CR. Adverse events were retrospectively identified from the medical records and graded according to the Common Terminology Criteria for Adverse Events version 5.0. Data were analyzed using descriptive statistics; continuous variables were summarized with medians and ranges, and categorical variables were summarized with frequencies and proportions. Frequency of objective responses was separately summarized for patients with multisystemic and/or solid lesions and for patients treated for ND-LCH. Overall survival was estimated with the Kaplan–Meier method.

## Results

### Patient and disease characteristics

**Figure 1.**
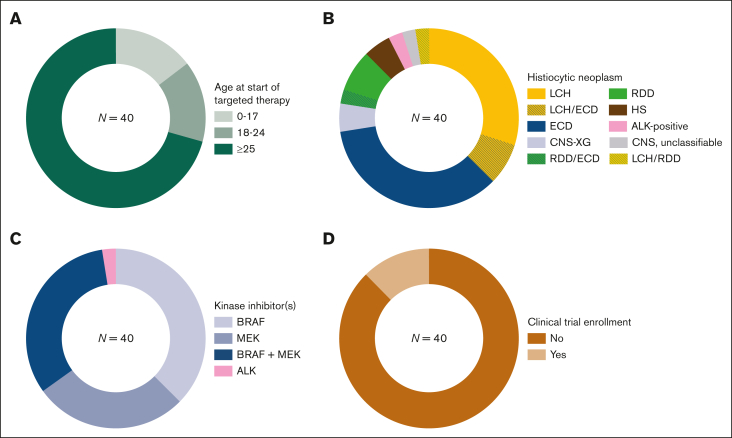
**Patient and treatment characteristics.** Pie charts depicting proportions of patients by (A) age category (with age measured in years at start of targeted therapy), (B) histiocytosis subtype, (C) inhibitor subtype (at start of targeted therapy), or (D) clinical trial enrollment.

A total of 40 patients with histiocytosis treated with targeted therapy were included. These comprised of 6 (15%) children (<18 years) and 34 (85%) adults at the time of targeted therapy initiation (Figure 1A), with a median age of 7.4 years (range, 0.6-9.8) among children and 52.6 years (range, 19.6-82.4) among adults. Patients were diagnosed with diverse histiocytic neoplasms (Figure 1B; Table 1), including LCH (n = 12), ECD (n = 14), isolated CNS xanthogranuloma (CNS-XG; n = 2), Rosai–Dorfman disease (RDD; n = 3), HS (n = 2), ALK-positive histiocytosis (n = 1), mixed LCH/ECD (n = 3), mixed LCH/RDD (n = 1), mixed RDD/ECD (n = 1), and unclassifiable CNS histiocytosis (n = 1). Of the 12 patients with LCH, 8 (67%) were treated for ND-LCH. In total, 12 patients had ND lesions at targeted therapy initiation, who all had LCH and/or ECD. The most commonly involved organs at the start of targeted therapy were the nervous system (20/40), bones (19/40), kidneys (14/40), skin (12/40), and cardiovascular system (11/40) (supplemental Table 1). Detected somatic genetic alterations included *BRAF*^*V600E*^ in 27 of 40 patients, *BRAF* exon 12 deletions in 5 of 40, *MAP2K1* mutations in 3 of 40, *NRAS/KRAS* mutations in 3 of 40 (including 1 with additional *BRAF*^*V600E*^), and *KIF5B*::*ALK* in 1 of 40. In 2 patients, no oncogenic alterations were detected (Table 1). The *MAP2K1* mutations in cases 13 and 30 were Raf-regulated (class II) mutations, known to be sensitive to allosteric MEK inhibitors in vitro*.*^59^ Case 38 had 2 *MAP2K1* alterations, including 1 missense mutation in the region where deletions are known to activate MEK1 in a Raf-independent manner.^59^

**Table 1.** **Clinical and molecular characteristics and treatment outcomes of included patients**

Case	Age at diagnosis	Sex	Histiocytic/dendritic cell neoplasm	Detected genetic alteration(s) in oncogenes∗	Disease extent at diagnosis	Number of prior Tx (number of chemo Tx)	Time from diagnosis to targeted Tx initiation	Disease extent at targeted therapy initiation	Inhibitor(s) at start	Clinical trial enrollment	Best response to targeted therapy	Time on inhibitor(s)	Targeted therapy ongoing at last FU
**LCH**													
1	5 mo	M	LCH	*BRAF* p.V600E	MS-RO–	4 (4)	20.6 y	ND-LCH	V	No	SD	0.6 y	Yes (V)
2	14 mo	F	LCH	*BRAF* p.V600E	MS-RO+	2 (2)	0.3 y	MS-RO+	D + T	No	CR	2.3 y	Yes (D)
3	2 y	M	LCH	*BRAF* p.V600E	SS-MFB	7 (3)	28.3 y	ND-LCH	V	No	Not evaluated	0.3 y	No
4	23 mo	F	LCH	*BRAF* p.V600E	SS-UFB	1 (1)	18.3 y	ND-LCH or MS-RO−†	T	No	SD (ND-LCH)‡	0.6 y	No
5	2 y	F	LCH	*BRAF* p.V600E	MS-RO−	2 (2)	6.4 y	ND-LCH	D + T	No	SD	1.9 y	Yes (D)
6	6 y	F	LCH	*BRAF* p.V600E §	ND-LCH	0 (0)	1.6 y	ND-LCH	D + T	No	SD (clinical); PR (radiologic)	2.3 y	Yes (D + T)
7	18 y	M	LCH	*BRAF* p.V600E	MS-RO−	4 (3)	5.6 y	ND-LCH	V	No	SD	0.5 y	No
8	19 y‖	M	LCH	*BRAF* p.N486_P490del	MS-RO−	2 (2)	4.7 y	MS-RO−	D + T	No	PR	0.2 y	Yes (D + T)
9	23 y	M	LCH	*BRAF* p.V600E	MS-RO−	1 (1)	3.6 y	ND-LCH	V	No	Not evaluated	1.5 mo + 1 mo	No
10	32 y	M	LCH	*BRAF* p.N486_P490del	MS-RO−	1 (1)	0.8 y	ND-LCH or MS-RO−¶	D + T	No	PR (ND-LCH); CR (skin)	0.6 y	Yes (D + T)
11	53 y	M	LCH	*BRAF* p.V600E	MS-RO−	1 (0)	4.1 y	MS-RO−	D + T	No	CR	0.7 y	Yes (D + T)
12#	53 y	F	LCH	*BRAF* p.N486_P490del	MS-RO+	0 (0)	0.9 y	MS-RO+	D	No	CR	2.0 y	No
**ECD**													
13∗∗	6 y††	F	ECD	*MAP2K1* p.E203K	MS	0 (0)	12 d	MS	T	No	CR	4.0 y	Yes (T)
14	22 y	M	ECD	*BRAF* p.V600E	MS	0 (0)	0.2 y	MS (with ND lesions)	V	No	PR (tumorous); SD (ND)	1.9 y	No
15	43 y	M	ECD	*BRAF* p.V600E	MS	0 (0)	0.2 y	MS	D	No	PR	2.4 y	Yes (D + T)
16	43 y	F	ECD	*BRAF* p.V600E	MS	1 (0)	2.3 y	MS	D	No	PR	5.2 y	Yes (T)
17	47 y‡‡	F	ECD	*BRAF* p.V600E & *KRAS* p.K117N	MS	0 (0)	0.2 y	MS (with ND lesions)	D + T	No	PR (tumorous and ND)	5.8 y	Yes (D+T)
18	51 y	M	ECD	*BRAF* p.V600E	SS	1 (0)	17.3 y	SS (kidney)	D + T	No	Not evaluated	18 d	No
19	54 y	M	ECD (with CMML-0)	*KRAS* p.G12A	MS	3 (0)	8.5 y	MS	T	No	PR	0.4 y	Yes (T)
20	56 y	M	ECD	*BRAF* p.V600E	MS	1 (0)	<1 mo	MS	V + Co	Yes: DRUP	Not reportable		
21	59 y	F	ECD	*BRAF* p.V600E	MS	2 (0)^a^	0.2 y	MS (with ND lesions)	D	No	PR (tumorous); SD (ND)	3.9 y	Yes (D + T)
22	67 y	M	ECD	*BRAF* p.V600E	MS	2 (0)^a^	0.8 y	MS	D	No	PR	2.7 y	Yes (T)
23	69 y	M	ECD	*BRAF* p.V600E	MS	0 (0)	1.6 y	MS	D + T	No	PR	3.6 y	Yes (D)
24	76 y	M	ECD	*BRAF* p.V600E	MS	1 (0)	0.6 y	MS	V + Co	Yes: DRUP	Not reportable		
25	77 y	F	ECD (with CML)	BRAF p.V600E	MS	0 (0)	0.2 y	MS	D^b^	No	PR	1.3 y	No
26	78 y	M	ECD	*BRAF* p.V600E	MS	1 (0)	3.8 y	MS	T	No	SD	0.5 y	No
**Xanthogranuloma of the CNS**													
27	6 m	F	CNS-JXG	*BRAF* p.V600E	SS	1 (0)	0.1 y	SS (spinal cord)	D + T	No	CR	2.4 y	Yes (D)
28	50 y	F	CNS-AXG	*BRAF* p.V600E	SS	3 (1)	1.7 y	SS (brain and spinal cord)	V	No	PR	0.5 y + 3.2 y	Yes (V)
**Mixed histiocytosis**													
29	46 y	M	LCH/RDD	*BRAF* p.N486_P490del	MS-RO+	2 (0)^a^	0.1 y	MS-RO+	Co	No	PR	5 wk	No
30	52 y	F	RDD/ECD	*MAP2K1* p.K57N	MS	5 (0)^a^	0.2 y	MS	Co	No	PR	1.8 y	No
31	53 y	M	LCH/ECD	*BRAF* p.V600E	MS-RO−	1 (1)	8.5 y	MS-RO−	D + T	No	PR	3.1 y	No
32	57 y	F	LCH/ECD	*BRAF* p.V600E	MS-RO−	2 (1)	9.4 y	MS-RO− (with ND lesions)	V	No	PR (tumorous)^c^	0.4 y + 1.9 y	No
33	65 y	M	LCH/ECD (with PCD)	*BRAF* p.V600E	MS-RO−	3 (2)	3.7 y	MS-RO−	V	No	PR (tumorous); PD (new ND)	3.5 y	No
**RDD**													
34	27 y	M	RDD	None (by targeted NGS)	MS	0 (0)	1.8 y	MS	Co	Yes: COBRAH	Not reportable		
35	42 y	F	RDD	None (by targeted NGS)	MS	4 (2)	3.2 y	MS	T	No	PR	1.7 y	Yes (T)
36	66 y	M	RDD (with PCD)	*BRAF* p.N486_P490del	SS	2 (1)	0.7 y	SS (brain and spinal cord)	Co	No	PR	3.3 y	Yes (Co)
**HS**													
37	19 y	M	HS (after B-ALL)	*NRAS* p.G12V^d^	SS	1 (1)^e^	0.2 y	SS (bone)	T^b^	No	CR	0.3 y	No
38	56 y	M	HS (primary)	*MAP2K1* p.F53L and p.E102G	MS	2 (2)	0.3 y	MS	T	Yes: DRUP	Not reportable		
**ALK-positive histiocytosis**													
39	9 y	F	ALK+ histiocytosis	*KIF5B*::*ALK* fusion	SS	1 (0)^a^	10 d	SS (soft tissue)	Cr	Yes: CRISP	Not reportable		
**CNS-histiocytosis, unclassifiable**													
40	52 y	F	Histiocytosis, UC	*BRAF* p.V600E	SS	1 (0)	0.1 y	SS (brain, CN V, pituitary)	V	No	PR	1 y + 0.8 y	Yes (V)

AXG, adult XG; CN V, trigeminal nerve; Co, cobimetinib; Cr, crizotinib; CML, chronic myeloid leukemia; CMML, chronic myelomonocytic leukemia; D, dabrafenib; FU, follow-up; F, female; JXG, juvenile XG; M, male; MFB, multifocal bone; MS, multisystem; PCD, plasma cell dyscrasia; RO, risk organ; SS, single system; Tx, therapies; T, trametinib; UC, unclassifiable; UFB, unifocal bone; V, vemurafenib.

∗ Given that different molecular assays were used for detection of genetic alterations, it cannot be excluded that (rare) genetic variants have been missed.

† In addition to neurodegenerative lesions, this patient potentially had an orbital lesion. The orbital lesion was surgically excised; histopathologic analysis could not confirm LCH involvement.

‡ MRI revealed mild progressive cerebellar atrophy. The orbital lesion was surgically excised; therefore, response to targeted therapy is not applicable.

§ In this patient with brain lesions characteristic of ND-LCH, droplet digital polymerase chain reaction analysis of DNA extracted from blood leukocytes detected a *BRAF*^*V600E*^ mutation.

‖ This patient had diabetes insipidus since 2013, and later panhypopituitarism. However, the diagnosis of LCH was made in 2018, when organs other than the pituitary (bone, skin, and lungs) became involved.

¶ This patient potentially had residual skin lesions. All other lesions present at diagnosis had resolved at the time of onset of ND-LCH and start of targeted therapy.

# This is patient 4 from a study by Renier et al.^60^

∗∗ This patient is reported in a study by Pegoraro et al.^61^

†† One year before ECD diagnosis (at the age of 5 years), this patient was diagnosed with multifocal skin JXG.

‡‡ Eight years before the diagnosis of ECD (at 38 years of age), this patient already had a thickened pituitary stalk.

a All or some of these treatments were given before the diagnosis of a histiocytic neoplasm was made.

b These 2 patients also received dasatinib for their second hematologic malignancy, including *BCR*::*ABL1*–positive CML (patient 25) or B-ALL with high *PDGFRB* expression by RNA sequencing (patient 37).

c In this patient, response of neurodegenerative lesions was not evaluated radiologically. However, neurologic symptoms (including dysarthria) appeared to slightly improve during targeted therapy, although this was not substantiated with repeated neurologic assessments using validated rating scales for neurologic symptoms.

d In tissue samples taken after disease progression despite chemotherapy, subclonal *BRAF*^*G469R*^ (calcaneus), *BRAF*^*D594N*^ (iliac bone), and *MTOR*^*S2215Y*^ (iliac bone) mutations were also detected.

e This does not include the prior treatments for the patient's B-ALL, before the diagnosis of secondary HS. For the initial B-ALL, this patient received chemotherapy (ALLTogether NCI HR protocol), followed by immunotherapy (2× CD19-directed CAR-T cell infusions; later 2 courses of 4 weeks of blinatumomab). The patient never achieved minimal residual disease negativity by polymerase chain reaction analysis of immunoglobulin gene rearrangements; the lowest minimal residual disease value was 0.02% at 6 weeks after first CAR-T cell infusion.

In addition to histiocytosis, 5 patients had another hematologic neoplasm (Table 1), including 2 with a kappa-monotypic plasma cell dyscrasia and single cases with chronic myeloid leukemia, chronic myelomonocytic leukemia, or B-cell acute lymphoblastic leukemia (B-ALL). The B-ALL preceded the diagnosis of HS, whereas the other neoplasms were diagnosed after the diagnosis of ECD, RDD, or mixed LCH/ECD. In 2 patients (patient 19 with ECD/chronic myelomonocytic leukemia and patient 37 with B-ALL/HS), identical *NRAS/KRAS* mutations were identified in both neoplasms, suggesting a clonal relationship.^4^ In the other 3, molecular analysis of shared genetic alterations either failed (patient 25), was not performed (patient 36), or was inconclusive (patient 33). In the latter, *BRAF*^*V600E*^ was detected (in 7/57 reads) in the bone marrow biopsy with 5% kappa-monotypic plasma cells and no histologic evidence of LCH/ECD; however, because of the low percentage of plasma cells, it could not be concluded with certainty that the plasma cells harbored the mutation.

### Treatment characteristics

Targeted therapy was initiated between 2015 and 2023; 26 of 40 (65%) patients started inhibitors in 2020 or later. Before targeted therapy, patients had received up to 7 lines of therapy (median, 1), which included up to 4 lines of chemotherapy (Table 1). Details of prior therapies are provided in supplemental Table 1. Most patients received targeted therapy as second- or further-line treatment; 9 of 40 (22.5%) patients received inhibitors as first-line therapy, including 6 with ECD and individual patients with ND-LCH, multisystem LCH, or multisystem RDD (Table 1). Patients were treated with diverse targeted agents, which was influenced by (1) the mutational status of their histiocytic neoplasms, (2) available clinical trials, and (3) varying connections between health care providers and pharmaceutical companies to arrange off-label targeted treatment. At initiation, targeted therapy consisted of vemurafenib in 9 (22.5%) patients, dabrafenib in 6 (15%), trametinib in 7 (17.5%), cobimetinib in 4 (10%), dual dabrafenib/trametinib in 11 (27.5%), dual vemurafenib/cobimetinib in 2 (5%), and crizotinib in 1 (2.5%). Thus, 15 (37.5%) patients received a BRAF inhibitor, 11 (27.5%) received a MEK inhibitor, 13 (32.5%) received both, and 1 (2.5%) received an ALK inhibitor at start of targeted therapy (Figure 1C). Only 5 of 40 (12.5%) patients received targeted therapy in the context of a clinical trial (Figure 1D), including 3 adults enrolled in the DRUP study (NCT02925234), 1 adult in the COBRAH study (NCT04007848), and 1 child in the CRISP study (EudraCT 2015-005437-53, ITCC-053). Outcome data of these 5 patients will be reported by the respective trials. Among the 35 patients treated with BRAF/MEK inhibitors outside of trials, median follow-up since the start of targeted therapy was 2.4 years (range, 0.1-7.2 years). Median time on targeted treatment was 1.9 years (range, 0.04-5.8 years). Twenty-seven (77%) patients received targeted therapy for multisystemic and/or solid lesions; 8 of 35 (23%) were treated for ND-LCH.

### Response of multisystemic and/or solid lesions

**Figure 2.**
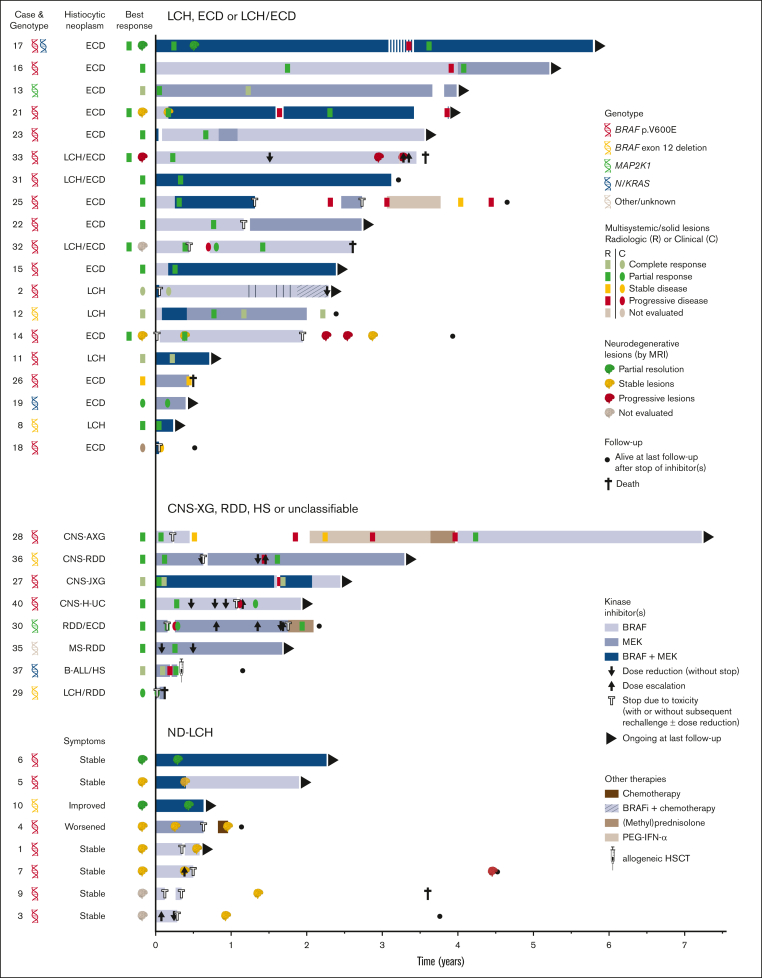
**Swimmer plot depicting targeted therapy outcomes.** Patients are grouped by disease subtype. For each patient, targeted therapy is represented by horizontal bars, with the size of these bars proportional to the length of treatment, and the color representing the type of treatment. Targeted therapy was initiated at time point zero. To the left of the horizontal bars, best response on targeted treatment is summarized for each patient; when applicable, separately for multisystemic/solid lesions and neurodegenerative lesions. Within the bars, outcomes of radiologic response assessments are shown as colored rectangles. In the absence of radiologic response assessments, clinical responses are visualized as colored ovals. In general, the first assessment establishing a particular response (eg, a PR) is depicted. AXG, adult xanthogranuloma; BRAFi, BRAF inhibitor; H-UC, unclassifiable histiocytosis; HSCT, hematopoietic stem cell transplantation; JXG, juvenile xanthogranuloma; MS, multisystem; PEG-IFN-α, pegylated interferon-α.

**Figure 3.**
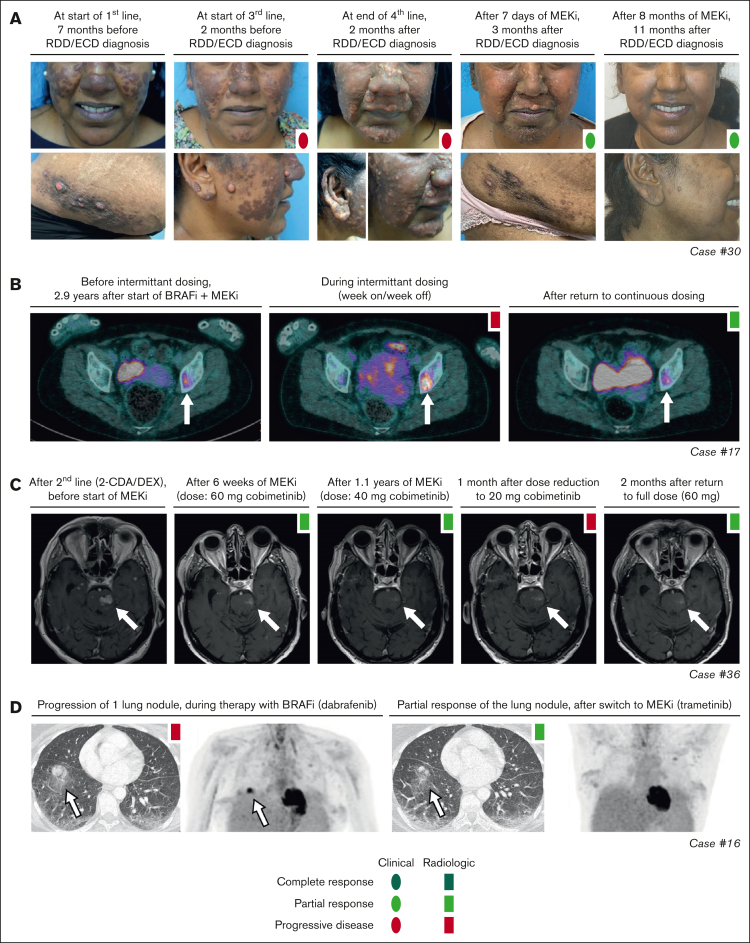
**Response and rare disease progression on targeted therapy.** (A) Photographs showing response of skin lesions in case 30 with RDD/ECD to conventional therapies and targeted treatment with cobimetinib. (B) Axial PET-computed tomography (CT) images of case 17 with ECD showing increased fluorodeoxyglucose (FDG) uptake of a left acetabular lesion (indicated by a white arrow) during intermittent dosing of dabrafenib/trametinib. After returning to continuous therapy, PET-CT showed decreased FDG uptake in the acetabular lesion, consistent with a recaptured PR. (C) Axial T1-weighted gadolinium-enhanced MRI images of case 36 with CNS-RDD showing an initial PR of contrast-enhanced brain lesions to treatment with cobimetinib, and subsequent progression of brain stem lesions after dose reduction to 20 mg/d. After return to full dose (60 mg/d), lesions decreased again, indicating a recaptured response. (D) Axial CT and PET images of case 16 with ECD showing progression of an FDG-avid lung nodule during treatment with dabrafenib, and partial resolution of this nodule after switch to treatment with trametinib. 2-CDA, cladribine; DEX, dexamethasone; BRAFi, BRAF inhibitor; MEKi, MEK inhibitor.

Objective responses were observed in 25 of 27 (93%) patients (Figure 2), whereas the remaining 2 patients either had SD (patient 26) or stopped treatment before response evaluation was possible (patient 18). Six patients obtained a CR, including 3 with LCH and individual patients with ECD, CNS-XG, or HS. Notably, the patient with HS (patient 37) stopped trametinib after 3.5 months, received an allogeneic hematopoietic stem cell transplantation, and was in complete remission at last follow-up (Figure 2). Objective responses were substantiated by imaging in 22 of 25 cases, and exclusively based on clinical and/or laboratory evaluations in the remaining 3 cases. For example, CR in case 2 (a 1-year-old girl with high-risk multisystem LCH) was based on marked clinical improvement and normalization of hemoglobin, platelet, and albumin levels. In case 30 with RDD/ECD, the objective response was also evident from rapid clinical improvement of skin lesions within days after start of cobimetinib (Figure 3A), with response of internal lesions captured by imaging after 8 weeks of treatment. Finally, the radiographic response in case 16 with ECD involving the lungs corresponded to a marked improvement of pulmonary function (transfer factor for carbon monoxide from 22% to 45%) and self-reported quality of life.

**Figure 4.**
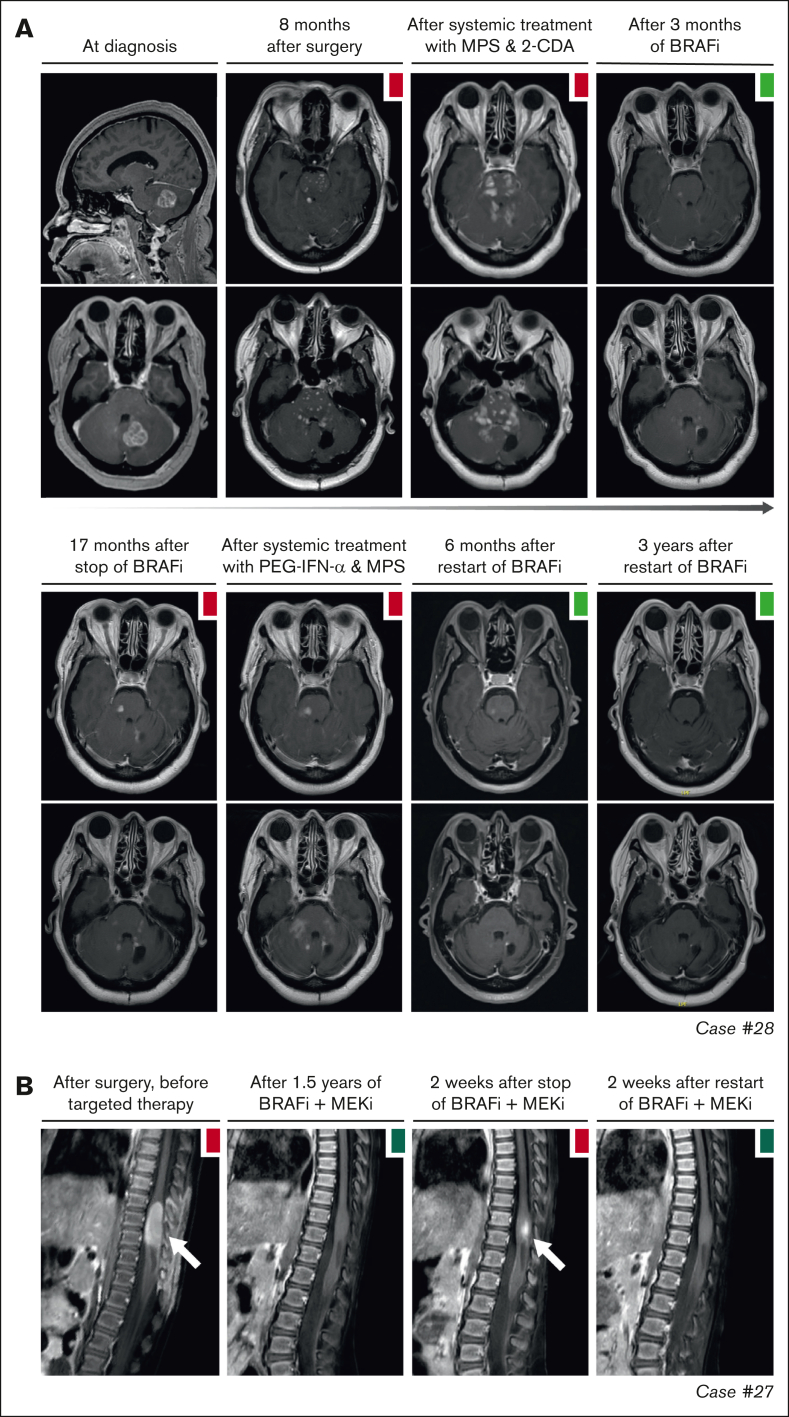
**Disease progression after targeted therapy interruption.** (A) Sagittal and axial MRI images of case 28, with CNS-AXG showing response of brain lesions to conventional therapies and targeted treatment with vemurafenib. After an initial PR to vemurafenib, targeted treatment was interrupted because of discontinued insurance coverage. Hereafter, brain lesions slowly progressed. Vemurafenib was restarted 3.6 years after interruption and quickly resulted in a decrease of brain lesions, with almost no lesions discernible at last follow-up. (B) Sagittal MRI images of case 27, with CNS-JXG showing relapse of a contrast-enhanced intramedullary spinal cord tumor after targeted therapy interruption. In this young child, treatment with dabrafenib and trametinib was interrupted after 1.6 years of therapy, while in complete radiologic remission. As an exit strategy, trametinib was stopped first, followed by dabrafenib 2 weeks later. MRI after stop of trametinib revealed persistent radiologic CR. However, MRI 2 weeks after stop of dabrafenib revealed recurrence of contrast-enhancement, consistent with a relapse of disease. MRI 2 weeks after restart of dabrafenib and trametinib showed that the radiologic response was recaptured. JXG, juvenile XG; MPS, methylprednisolone.

Objective responses were generally durable, although there were instances of PD in 11 of 27 (41%) patients (Figure 2). In 10 of 11 patients, these instances were related to a dose reduction or therapy interruption. Responses were recaptured in 9 of 10 cases after the dose was increased or therapy was restarted. For example, case 17 with ECD had increased fluorodeoxyglucose uptake in a left acetabular lesion after intermittent dosing of dabrafenib/trametinib (week on/week off; initiated as exit strategy after 3 years of treatment). After returning to continuous therapy, fluorodeoxyglucose uptake in the acetabular lesion decreased again (Figure 3B). Similarly, in case 36 with CNS-RDD (Figure 3C) and cases 27 and 28 with CNS-XG (Figure 4), disease progression was observed on MRI after dose reduction or stop of targeted therapy. In all 3 cases, objective responses were recaptured after dose escalation or restart of therapy (Figure 2). Potential acquired resistance to targeted therapy was only observed in case 16 with ECD, who had progression of a single lung nodule despite adequate intake and dosing of dabrafenib (150 mg twice daily). No biopsy of the nodule was taken. The patient subsequently switched to trametinib, which resulted in a PR at the end of follow-up (Figure 3D).

### Response and progression of ND lesions

**Figure 5.**
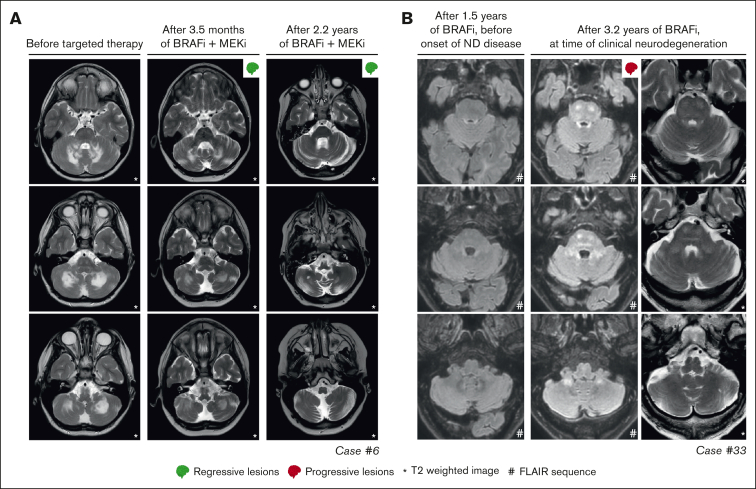
**Response and progression of ND lesions.** (A) Axial MRI images of case 6, with isolated ND-LCH showing significant reduction in T2-hyperintense lesions after treatment with dabrafenib and trametinib. Note that the remaining hyperintense lesion in the right cerebellum at last follow-up (right column; middle image) is related to the biopsy that was taken from this anatomical site. (B) Axial MRI images of case 33 showing the development of T2- and FLAIR-hyperintense lesions in the pons and cerebellum while receiving treatment with vemurafenib. The patient also developed clinical symptoms of neurodegeneration and died 7.5 months after the MRI scan depicting ND lesions was made. ND, neurodegenerative; FLAIR, fluid-attenuated inversion recovery.

In 4 of 27 patients with multisystemic and/or solid lesions, brain lesions characteristic of histiocytosis-associated neurodegeneration were present on MRI at targeted therapy initiation. Only 1 of 4 also had clear neurologic symptoms (patient 32 with dysarthria). During targeted therapy, radiologic response of the brain lesions was noted in case 17 (supplemental Figure 1A), whereas lesions remained stable in 2 of 4 and were not evaluated in case 32, who appeared to have a slight improvement in dysarthria. One of the patients with stable brain lesions during targeted therapy (patient 14 with ECD) had progressive radiologic abnormalities in the pons and cerebellum at 4 and 7 months after stopping vemurafenib because of toxicity (supplemental Figure 1B). This patient did not have progressive neurologic symptoms; therefore, no treatment was initiated. At 2 years after stopping vemurafenib, the patient’s clinical and radiologic situation remained stable. Another patient (case 33 with LCH/ECD) developed new radiologic abnormalities in the pons and cerebellum and clinical symptoms of neurodegeneration after almost 3 years of treatment with vemurafenib (Figure 5B). The patient subsequently received (methyl)prednisolone and increased doses of vemurafenib (up to twice daily 720 mg; 4 out of 6 days), without a clinical response. Eventually, targeted treatment was switched to dabrafenib, but the patient requested euthanasia a few weeks later and died shortly thereafter.

Among the 8 patients receiving targeted therapy for ND-LCH, 2 had a PR of radiologic abnormalities (Figure 5A), 4 had stable lesions, and 2 stopped therapy before response could be evaluated (Figure 2). The 2 patients with a response were treated within 2 years after diagnosis of LCH, whereas the 4 with stable lesions started targeted therapy between 5 and 20 years after diagnosis of LCH. The radiologic response was accompanied by improvement of cognitive symptoms of memory impairment and concentration difficulties in case 10 (who did not have ataxia/dysarthria), whereas repeated neurologic assessments in case 6 showed a rather stable situation, with only slightly improved International Cooperative Ataxia Rating Scale scores (45 to 42).^62^

### Toxicity

Adverse events were observed in 26 of 35 (74%) patients, although most were grade 1 to 2 events that required no or minimal intervention (supplemental Table 2). Skin-related adverse events were most common (supplemental Figure 2). Eight patients had grade 3 events, including a wide range of clinical symptoms/conditions, such as gastrointestinal hemorrhage (patient 2; dabrafenib/trametinib), oral mucositis (patient 4; trametinib), uveitis (patient 16; dabrafenib), pneumonitis (patient 30; cobimetinib), and photosensitivity (patient 32; vemurafenib). No grade 4 events were recorded. One patient (case 29) with severe LCH-related liver failure had a grade 5 duodenal hemorrhage, which could have been related to treatment with prednisone and/or cobimetinib.

Because of toxicity, targeted therapy was interrupted in 16 of 35 (46%) patients. Treatment was often resumed after a treatment-free interval (“drug holiday”), with or without dose reduction (Figure 2). At last follow-up, 20 of 35 (57%) patients remained on targeted therapy, whereas 8 of 35 (23%) had stopped because of toxicity, 2 of 35 (6%) had stopped because of medical procedures/unrelated symptomatology, 2 of 35 (6%) had stopped because of lack of response (patient 26 with SD; patient 33 with progressive ND disease), 2 of 35 (6%) had died while on targeted therapy (despite a response), and 1 had stopped because of subsequent allogeneic hematopoietic stem cell transplantation (Figure 2; supplemental Table 1). All 8 patients who stopped because of toxicity were adults.

### Deaths

**Figure 6.**
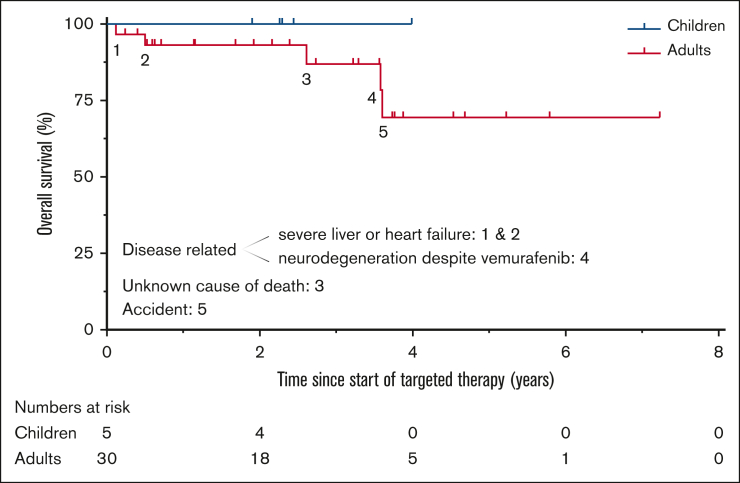
**Overall survival and causes of death.** Kaplan–Meier curves depicting overall survival for children and adults treated with targeted therapy outside clinical trials. Five of the 35 patients died, including 3 from disease-related causes; “1” is case 29, “2” is case 26, “3” is case 32, “4” is case 33, and “5” is case 9.

Five patients died during follow-up, including 3 from disease-related causes, 1 from an accident, and 1 from unknown cause (Figure 6). Disease-related causes included progressive ND disease (patient 33), ECD-related heart failure (patient 26), and complications of LCH-related liver failure and/or treatment with prednisone and cobimetinib (patient 29 with a grade 5 duodenal hemorrhage). Details are provided in supplemental Table 1.

## Discussion

We report real-world experience with targeted therapy in the treatment of patients with histiocytic neoplasms. Objective responses were observed in 25 of 27 (93%) patients with multisystemic and/or solid lesions, who were treated with diverse BRAF and/or MEK inhibitors. Responses were irrespective of mutational profile and observed across histiocytic neoplasms. Furthermore, partial resolution of MRI abnormalities was observed in 2 of 8 (25%) patients treated for ND-LCH, with clinical improvement in 1 of these cases.

Our data corroborate the favorable outcomes of BRAF/MEK inhibition in patients with histiocytosis described previously.^18^^,^^47^^,^^51^^,^^55^^,^^63^, ^64^, ^65^, ^66^, ^67^ In a phase 2 study of 18 adults with histiocytosis treated with cobimetinib, the overall response rate was 89%.^18^ Moreover, in studies of children with multisystem LCH treated with BRAF/MEK inhibitors, response rates ranged between 58% and 100%.^51^^,^^64^^,^^66^ Our study also demonstrates responses in rare histiocytosis subtypes, such as RDD, isolated CNS-XG, and HS. Specifically, case 37 with HS achieved complete remission, which is exceptional for this disease with an otherwise dismal outcome.^30^^,^^68^, ^69^, ^70^ Yet, it should be noted that this patient had single-system bone disease secondary to B-ALL, which is different from primary HS. Our findings regarding ND-LCH contrast with a previous study that reported a high response rate of 92% in 13 patients with the condition.^65^ However, we need to point out that the 8 patients from our study generally had short treatment durations (median, 0.6 years). In addition, targeted therapy was initiated years after disease onset in patients without a response. In these cases, neurologic damage may have already become irreversible. Therefore, we regard the response in a subset of our patients as significant, considering that conventional therapy is rarely effective in treating ND-LCH.^29^

Despite the favorable responses, our study shows that targeted therapy is not a perfect remedy. One limitation is toxicity, leading to stop of targeted therapy in almost a quarter of our patients, all of whom were adults. Previous studies have also noted that adults with histiocytic neoplasms appear particularly prone to toxicity, both in relation to conventional and targeted therapies.^56^^,^^71^ Dose reductions or “drug holidays” could often reduce toxicity to acceptable levels and prevent discontinuation of targeted treatment. However, they were also associated with the development of PD in some of our patients. Thus, finding the right balance between drug tolerability and efficacy can be challenging.^72^

Another limitation of targeted therapy is that it is often not capable of eradicating the histiocytic disease. Among the 25 patients with an objective response of their multisystemic and/or solid lesions, 19 (76%) had a PR. Both in patients with a PR or CR, stop of targeted therapy can quickly result in relapse of the disease.^63^^,^^64^^,^^73^ Nevertheless, some patients may be eligible for limited-duration treatment, as a recent institutional cohort study reported 5 adult patients with diverse histiocytic neoplasms who did not experience disease relapse following discontinuation of targeted therapy.^73^ Similarly, another study described 5 children with single system bone LCH who discontinued targeted therapy without recurrence, although spontaneous remission cannot be excluded in such cases.^67^ In most patients, combinations of targeted and conventional therapies are probably required to eradicate the neoplastic clone,^74^ with encouraging results from a recent clinical trial combining vemurafenib with cladribine and cytarabine for the treatment of children with multisystem LCH.^75^ Less toxic regimens are presumably needed in adults.

Another potential limitation is that targeted therapy may not always be able to prevent the development of ND disease, as illustrated by case 33. Yet, this patient with LCH/ECD developed ND disease during treatment with vemurafenib (low-dose: twice daily 240 mg), which is known to have poor CNS penetration.^32^^,^^76^ Other inhibitors, such as the pan-Raf inhibitor tovorafenib (DAY101), which is currently being tested in a phase 2 trial for relapsed/refractory LCH (NCT05828069), are considered to have improved CNS penetration.^77^^,^^78^ Furthermore, combined BRAF/MEK inhibition might be superior to monotherapy for managing CNS disease, although supporting data are still limited.^76^ In a recent study by Cournoyer et al,^67^ 0 of 34 patients with histiocytosis treated with dabrafenib and/or trametinib developed neurodegeneration while on therapy, with a median treatment duration of 3.3 years. However, more studies with long-term data are needed to address the important issue of how to treat and prevent histiocytosis-associated neurodegeneration.^29^^,^^79^

Despite these limitations of targeted therapy, the availability of a new therapeutic option for patients with histiocytic neoplasms represents a breakthrough in an area of previously unmet medical need. Active matters of debate are the optimal patient population and timing of targeted therapy initiation. Although some histiocytosis experts advocate its use as first-line therapy in all patients with histiocytosis who require systemic treatment,^67^ most physicians restrict the treatment to patients with severe and/or relapsed/refractory disease. In our study, all 9 patients receiving BRAF/MEK inhibition as first-line therapy had severe (multisystem or ND) disease. The main argument for the use of inhibitors as first-line therapy are the considerable rates of disease progression/relapse with conventional therapy.^67^ Secondary benefits are ease of administration, avoiding toxicities of conventional therapies, and preventing irreversible disease-related organ damage.^67^ The main arguments against are the unknown long-term safety and efficacy of targeted therapy, as the first patient in the phase 1 trial of vemurafenib was enrolled in 2006^80^ and because insights into toxicity are not universally applicable across different disorders. For example, skin carcinomas are a common adverse event of vemurafenib in patients with melanoma,^81^ but have been rarely observed in patients with histiocytosis treated with this agent.^47^^,^^63^^,^^64^ In contrast, chemotherapeutic agents such as vinblastine have been used since the 1960s and are known to induce durable remissions with limited-duration treatment (eg, 12 months) in a substantial proportion of patients with histiocytosis.^23^ Considering their ongoing physical development and remaining life span, long-term safety is particularly relevant to the pediatric population. Recently, a decrease in growth velocity of children with low-grade glioma treated with the pan-Raf inhibitor tovorafenib was reported,^78^ warranting further evaluation. The crux is to identify patients who are at high risk of disease progression and/or permanent consequences so that they can receive early access to targeted therapies. Future studies should be directed at identifying these risk factors.

Other unresolved issues are the duration of targeted therapy and optimal combination strategies with other agents. Regarding duration, a highly sensitive minimal residual disease marker could help decide when to stop therapy.^67^ Accordingly, multiple groups have started measuring mutant *BRAF*^*V600E*^ alleles in cell-free DNA and/or DNA derived from blood or bone marrow cells.^65^^,^^67^^,^^82^, ^83^, ^84^, ^85^, ^86^, ^87^, ^88^, ^89^, ^90^, ^91^ These studies have revealed that mutant alleles often persist in the blood despite targeted therapy. However, more detailed studies investigating longitudinal patterns of mutant DNA alleles in large groups of patients with and without targeted therapy are needed to fully grasp the clinical implications. Another promising biomarker requiring further validation is neurofilament light chain in the blood or cerebrospinal fluid, which might aid in detecting and monitoring ND disease.^92^^,^^93^ Therefore, it is crucial that future prospective studies of patients with histiocytosis treated with targeted therapy are complemented by the collection of biological samples to facilitate companion biology studies.

Our study has several limitations, many of which relate to its retrospective design. Consequently, our patients have been treated with diverse targeted agents at varying doses, making it difficult to draw conclusions on specific inhibitors and doses. Furthermore, treatment decisions (ie, regarding dosing and dose modification) were made on the basis of clinician judgment rather than strict study protocols. In addition, organ involvement and response to treatment were not uniformly assessed but based on standard-of-care practices in different institutions. Finally, adverse events were not prospectively registered but retrospectively identified, which is susceptible to inaccuracies. Prospective studies are needed to demonstrate long-term safety and efficacy rigorously and to determine the optimal patient population, timing, dosage, and duration of targeted therapy. Unfortunately, few clinical trials currently exist, with limited support from the pharmaceutical industry for additional (histiocytosis-specific) trials.^67^ Given the strict eligibility criteria in clinical trials, real-world data studies remain essential to assess the value of inhibitors in the actual patient population; accordingly, we consider the diverse nature of our cohort as an important virtue of our study.

In conclusion, we show frequent objective responses to targeted therapy, but also rare instances of progressive ND lesions in patients treated with vemurafenib. Toxicity of targeted therapy was generally acceptable, although it caused a substantial number of adults to stop treatment. Moreover, multiple patients experienced disease relapse following dose reduction or therapy interruption. Prospective studies should assess novel inhibitors with improved CNS penetration and combination strategies of targeted and conventional agents to eradicate the neoplastic clone and thereby achieve cure of patients.

Conflict-of-interest disclosure: S.H.T. received financial compensation from Roche for a review of the American Society of Hematology Annual Meeting and Exposition. P.S. reports consulting/advisory roles for Deciphera, Ellipses Pharma, Transgene, Exelixis, Boehringer Ingelheim, Studiecentrum voor Kernenergie, Adcendo, PharmaMar, Merck Healthcare KGaA, Medpace, Cogent Biosciences, Eisai, Curio Science, LLX Solutions, Servier, Genmab, Sanofi, Regeneron, Moleculin Biotech, Avacta Life Sciences, Amryt Pharma, Union Chimique Belge, Boxer Capital, NEC OncoImmunity AS, Sonata Therapeutics, IDRx, and Telix Pharmaceuticals; and institutional research funding from CoBioRes NV, Eisai, G1 Therapeutics, PharmaMar, Genmab, Merck, Sartar Therapeutics, ONA Therapeutics, and Adcendo. The remaining authors declare no competing financial interests.

A complete list of the members of the Dutch-Belgian Cooperative Trial Group for Hematology Oncology Histiocytic and Lymphocytic Diseases Working Group appears in “Appendix.”

## Appendix

The complete list of the members of the Dutch-Belgian Cooperative Trial Group for Hematology Oncology Histiocytic and Lymphocytic Diseases working group are Erik Beckers (Dept. of Internal Medicine, Maastricht University Medical Center); Merlijn van den Berg (Emma Children’s Hospital, Amsterdam University Medical Centers); Cor van den Bos (Princess Máxima Center for Pediatric Oncology); Godelieve de Bree (Dept. of Internal Medicine, Amsterdam University Medical Centers); Emmeline Buddingh (Willem[1]Alexander Children’s Hospital, Leiden University Medical Center); Kristl Claeys (Dept. of Neurology, University Hospitals Leuven); Paul van Daele (Dept. of Internal Medicine, Erasmus Medical Center); Petra De Haes (Dept. of Dermatology, University Hospitals Leuven); Astrid Demandt (Dept. of Hematology, Maastricht University Medical Center); Suzanne van Dorp (Dept. of Hematology, Radboud University Medical Center); Liesbeth Hak (Dept. of Internal Medicine, Amsterdam University Medical Centers); Astrid van Halteren (Dept. of Internal Medicine, Erasmus Medical Center); Tim van der Houwen (Dept. of Internal Medicine, Amsterdam University Medical Centers); Margot Jak (Dept. of Hematology, University Medical Center Utrecht); Jeroen Kerstens (Dept. of Neurology, Antwerp University Hospital); Arjan Kwakernaak (Dept. of Internal Medicine, Amsterdam University Medical Centers); Jan van Laar (Dept. of Internal Medicine and Dept. of Immunology, Erasmus Medical Center); Helen Leavis (Dept. of Internal Medicine, University Medical Center Utrecht); Roos Leguit (Dept. of Pathology, University Medical Center Utrecht); Arjan van de Loosdrecht (Dept. of Hematology, Amsterdam University Medical Centers); Linde Morsink (Dept. of Hematology, University Medical Center Groningen); Rogier Mous (Dept. of Hematology, University Medical Center Utrecht); Max van Noesel (Princess Máxima Center for Pediatric Oncology); Rimke Oostvogels (Dept. of Hematology, University Medical Center Utrecht); Judith Potjewijd (Dept. of Internal Medicine, Maastricht University Medical Center); Wouter Plattel (Dept. of Hematology, University Medical Center Groningen); Wilfried Roeloffzen (Dept. of Hematology, University Medical Center Groningen); Abraham Rutgers (Dept. of Rheumatology and Clinical Immunology, University Medical Center Groningen); Sanne Tonino (Dept. of Hematology, Amsterdam University Medical Centers); Thomas Tousseyn (Dept. of Pathology, University Hospitals Leuven); Rob Verdijk (Dept. of Pathology, Erasmus Medical Center); Joost Vermaat (Dept. of Hematology, Leiden University Medical Center); Sherida Woei-A-Jin (Dept. of General Medical Oncology, University Hospitals Leuven).
