# Supplementary material for: Real-world experience with targeted therapy in patients with histiocytic neoplasms in the Netherlands and in Belgium
Source: Blood Neoplasia. 2024 Jun 10;1(3):100023. doi: 10.1016/j.bneo.2024.100023 (PMC12096411; doi:10.1016/j.bneo.2024.100023)
Supplement: Supplemental Figures and Tables [file BNEO_NEO-2024-000271-mmc1.pdf]

SUPPLEMENTARY FIGURE 1. Response and progression of neurodegenerative lesions

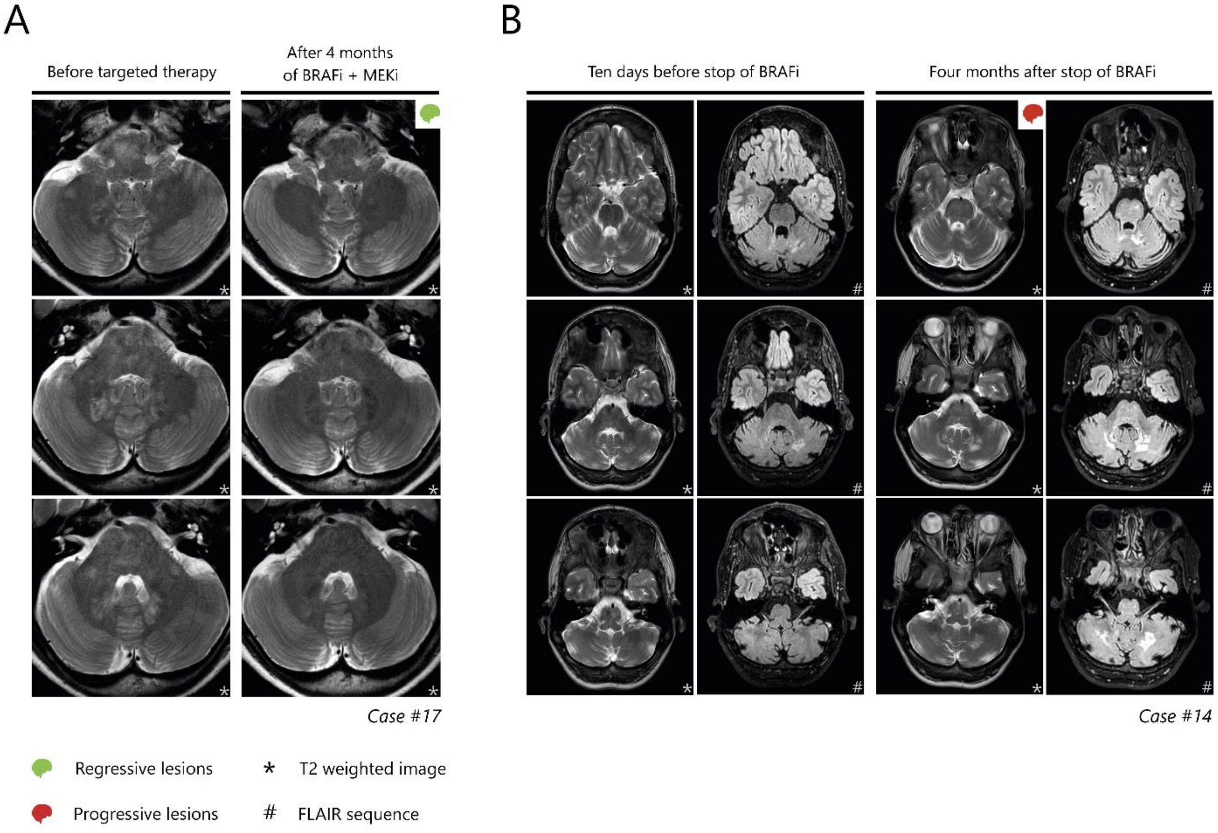

(A) Axial MRI images of Case #17 with ECD showing partial response of T2-hyperintense lesions in the brain stem and cerebellum after start of targeted therapy with dabrafenib and trametinib. **(B)** Axial MRI images of Case #14 with ECD showing increase in T2- and FLAIR-hyperintense lesions after stop of vemurafenib due to persistent toxicity. The patient did not experience an increase in neurologic symptoms; therefore, no treatment was initiated.

SUPPLEMENTARY FIGURE 2. Number of patients with adverse events by System Organ Class

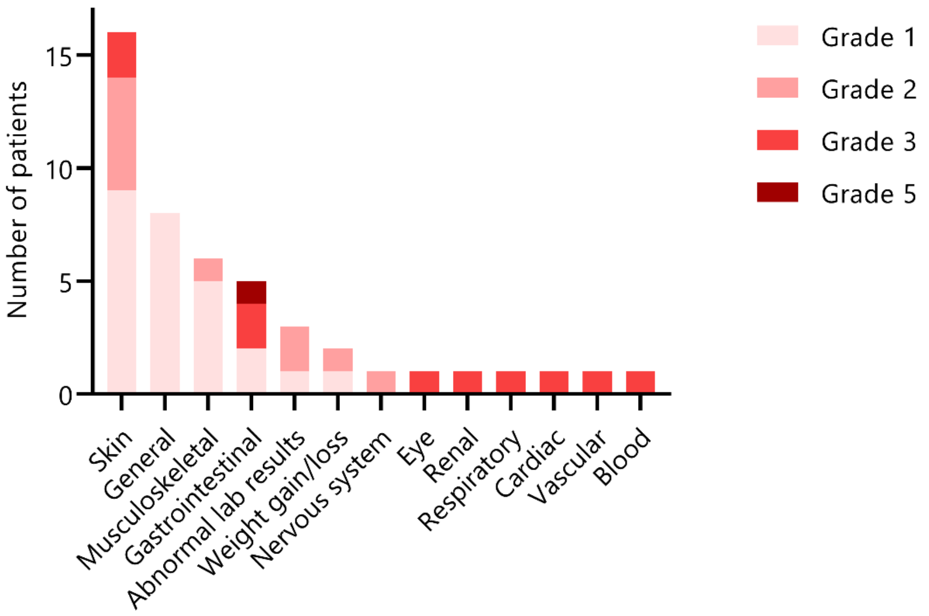

System Organ Class (SOC) and grade of adverse events were defined by the Common Terminology Criteria for Adverse Events (CTCAE) version 5.0. Within the SOC "Investigations", "Abnormal lab results" and "Weight gain/loss" were distinguished. If a patient had adverse events with different grades from a single SOC, the highest grade is depicted. Details of adverse events are provided in Supplementary Table 2.

SUPPLEMENTARY TABLE 1. Detailed disease characteristics and treatment data of included patients

Case	Type of prior treatment(s)	Last response to prior treatment	Disease extent at targeted therapy	Sites of disease at start of targeted therapy	Inhibitor(s) at start	Targeted therapy ongoing (current dose)	Reason for stop of targeted therapy	Conventional treatment started during or after targeted therapy	Status (follow-up since diagnosis)
**Langerhans cell histiocytosis**									
1	*For tumorous lesions*<br>1+2) VBL/MTX/6-MP/PRED (LCH-III protocol)<br>3) ARA-C/MTX/6-MP/PRED<br>4) 2-CDA/PRED (6 courses)	CR (tumorous lesions)	ND-LCH	Brain	V	Yes (twice daily 240mg)	-	No	Alive (21.2 y)
2	1) VBL/PRED (LCH-IV Stratum I, IC1, extended)<br>2) 2-CDA/ARA-C/DEX (1 course)[2]	PR	MS-RO+	Hematopoietic system, liver	D + T	Yes, D (twice daily 20mg)	Stop of T due to suspected toxicity (Grade III, gastrointestinal hemorrhage)	1) ARA-C (2x 5 days, subcutaneous)<br>2) 2-CDA/ARA-C/DEX (3 courses)[10]<br>3) VBL/6-MP/PRED (LCH-IV, Stratum I, Gr. 1, maintenance)	Alive (2.6 y)
3	*For tumorous lesions*<br>1) VBL/VP-16/PRED<br>2) Cryosurgery and locoregional steroids<br>3) Cyclosporine<br>4) VP-16/MPS (LCH-I protocol, arm B)<br>5) VBL/VP-16/MPS (LCH-I protocol, arm A+B)<br>6) Systemic corticosteroids (PRED)<br>*For neurodegenerative lesions*<br>7) IVIG	• CR (tumorous lesions)<br>• PD (ND-LCH)	ND-LCH	Brain	V	No	Toxicity (Grade III, skin toxicity: painful exanthema)	No	Alive (32.0 y)
4	*For tumorous lesions*<br>VBL/PRED	CR (tumorous lesions)	ND-LCH or MS-RO-[6]	Brain (ND-LCH)<br>*Possibly: bone (orbit)*[6]	T	No	No response & toxicity (Grade III, oral mucositis)	ARA-C (intrathecal; for ND-LCH)	Alive (19.4 y)
5	*For tumorous lesions*<br>1) VBL/PRED (LCH-IV Stratum I, IC1-2)<br>2) VCR/ARA-C/PRED (LCH-IV Stratum II)	CR (tumorous lesions)	ND-LCH	Brain	D + T	Yes, D (twice daily 75mg)	Stop of T due to suspected toxicity (Grade I, weight gain)	No	Alive (8.3 y)
6	-	-	ND-LCH	Brain	D + T	Yes, D (twice daily 110mg) + T (once daily 1mg)	-	No	Alive (3.9 y)
7	*For tumorous and neurodegenerative lesions*<br>1) ARA-C/PRED/IVIG<br>2) VBL/6-MP/PRED (LCH-A1 protocol)<br>3) 2-CDA<br>4) Anakinra	• CR (tumorous lesions)<br>• PD (ND-LCH)	ND-LCH	Brain	V	No	No response & toxicity (Grade II, skin toxicity: acneiform rash, decreased platelet count, fatigue, weight loss)	No	Alive (10.2 y)
8	1) VBL/PRED (LCH-IV Stratum I, 12 months)<br>2) MTX (max. 20mg/week, 18 months)	PD	MS-RO-	Bone, pituitary, skin	D + T	Yes, D (twice daily 150mg) + T (once daily 2mg)	-	No	Alive (4.9 y)
9	*For tumorous and neurodegenerative lesions*<br>2-CDA (6 courses); AZA maintenance	• CR (tumorous lesions)<br>• PD (ND-LCH)	ND-LCH	Brain	V	No	Toxicity (Grade I, flu like symptoms, arthralgia, fatigue)	No	Died with stable ND-LCH in an accident (7.2 y)
10	*For tumorous lesions*<br>VBL/PRED (LCH-A1 protocol)	PR (tumorous lesions)	ND-LCH or MS-RO-[7]	Brain (ND-LCH)<br>*Possibly: skin*[7]	D + T	Yes, D (twice daily 150mg) + T (once daily 2mg)	-	No	Alive (1.4 y)
11	Radiotherapy (24 Gy)	PD	MS-RO-	Bone, pituitary, skin	D + T	Yes, D (twice daily 150mg) + T (once daily 2mg)	-	No	Alive (4.8 y)
12 [1]	-	-	MS-RO+	Genital mucosa, GI tract, liver, pituitary, skin (incl. external ear), soft tissue (synovial nodules), thyroid	D	No	Stop on request of patient	No	Alive (3.3 y)

Case	Type of prior treatment(s)	Last response to prior treatment	Disease extent at targeted therapy	Sites of disease at start of targeted therapy	Inhibitor(s) at start	Targeted therapy ongoing (current dose)	Reason for stop of targeted therapy	Conventional treatment started during or after targeted therapy	Status (follow-up since diagnosis)
**Erdheim-Chester disease**									
13	-	-	MS	Bone, cardiovascular system, CNS (intraspinal dural masses), lymph node, soft tissue	T	Yes (once daily 0.25mg)	-	No	Alive (4.0 y)
14	-	-	MS (with ND lesions)	Bone, brain (ND lesions cerebellum), cardiovascular system, cavernous sinuses, kidney, orbital cavity, pituitary, soft tissue (thoracal epidural masses that extend into the spinal canal and neuroforamina), thymus/anterior mediastinum	V	No	Toxicity (Grade I-II, skin toxicity: photosensitivity, myalgia, arthralgia, fatigue, alopecia)	No	Alive (3.9 y)
15	-	-	MS	Bone, kidney, pituitary, skin	D	Yes, D (twice daily 150mg) + T (once daily 2mg)	-	No	Alive (2.6 y)
16	PEG-IFN-α	PD	MS	Bone, cardiovascular system, hematopoietic system, kidney, liver, lung, lymph node, skin, soft tissue (incl. breast), spleen	D	Yes, T (once daily 1.5mg)	Switch to T because of disease progression (lung nodule only)	No	Alive (7.5 y)
17	-	-	MS (with ND lesions)	Bone, brain (ND lesions pons and cerebellum), cardiovascular system, hematopoietic system, kidney, orbital cavity, pituitary, skin, soft tissue (retroperitoneal mass)	D + T	Yes, D (twice daily 150mg) + T (once daily 2mg)	-	No	Alive (6.0 y)
18	IFN-α, followed by PEG-IFN-α	PD	SS	Kidney	D + T	No	Toxicity (Grade I, fever, diarrhea); given limited disease, patient preferred to stop	No	Alive (17.9 y)
19	1) Steroids (MPS/PRED)<br>2) Steroids (MPS/PRED) + MTX (20-25mg)<br>3) Steroids (MPS/PRED) + Sirolimus, quickly switched to steroids (PRED) + PEG-IFN-α	PD	MS	Bone, skin	T	Yes (once daily 2mg)	-	No	Alive (8.9 y)
20	PEG-IFN-α	PD	MS	Bone, cardiovascular system, kidney, lung, orbital cavity, pancreas, spleen	V + Co		Not reportable		
21	1) MPS/AZA[3]<br>2) MPS/rituximab[3]	PD	MS (with ND lesions)	Brain (ND lesions cerebellum and pons), cardiovascular system, kidney, orbital cavity, pancreas, skin	D	Yes, D (twice daily 150mg) + T (once daily 2mg)	-	No	Alive (4.0 y)
22	1) MPS/tocilizumab[3]<br>2) MTX (max. 20mg/week)[3,4]	PD	MS	Bone, kidney	D	Yes, T (once daily 0.5mg)	Switch to T because of toxicity D (Grade I, myalgia, arthralgia)	No	Alive (3.5 y)
23	-	-	MS	Bone, cardiovascular system, kidney, orbital cavity, peripheral nerves (S1/S2 roots)	D + T	Yes, D (twice daily 50mg)	Brief stop of D + T because of low motivation to take medication; switch to monotherapy with D to improve treatment adherence	No	Alive (5.1 y)
24	Systemic corticosteroids (PRED)	PR	MS	Lung (pleura), soft tissue (thoracal mass between esophagus and vertebrae)<br>*Possibly: cardiovascular system, kidney, pituitary, spinal cord*	V + Co		Not reportable		
25	-	-	MS	Bone, cardiovascular system, kidney, orbital cavity<br>*Possibly: lung (pleura)*	D	No	Toxicity (Grade III, acute kidney injury, anemia) of combination trametinib with dasatinib. The latter was given for CML.	PEG-IFN-α	Alive (4.8 y)
26	IFN-α, followed by PEG-IFN-α	SD	MS	Bone, cardiovascular system, kidney, orbital cavity	T	No	Decision to pursue end-of-life care	No	Died with disease and severe ECD-related heart failure (4.3 y)

Case	Type of prior treatment(s)	Last response to prior treatment	Disease extent at targeted therapy	Sites of disease at start of targeted therapy	Inhibitor(s) at start	Targeted therapy ongoing (current dose)	Reason for stop of targeted therapy	Conventional treatment started during or after targeted therapy	Status (follow-up since diagnosis)
**Xanthogranuloma of the central nervous system**									
27	Subtotal surgical resection	PD	SS	Spinal cord (intramedullary tumor Th9-Th11)	D + T	Yes, D (twice daily 30mg)	Stop of T as monotherapy with D was also effective (shared decision parents & physician)	No	Alive (2.5 y)
28	1) Subtotal surgical resection<br>2) Systemic corticosteroids (MPS)<br>3) 2-CDA (2 courses), followed by MPS	PD	SS	Brain (tumorous lesions cerebellum, left and right of 4th ventricle, pons, occipital lobe, parietal lobe, and mesencephalon), spinal cord (intramedullary/leptomeningeal lesion at C5)	V	Yes (twice daily 480mg)	-	1) PEG-IFN-α<br>2) Systemic corticosteroids (MPS)	Alive (9.0 y)
**Mixed histiocytosis**									
29	1) Systemic corticosteroids (PRED)[3]<br>2) Liver transplantation[3]	PD	MS-RO+	Brain (tumorous lesions pons and medial temporal regions), liver, pituitary, soft tissue (leg)	Co	No	Death[9]	No	Died with disease and LCH-related liver failure from abdominal bleeding, sepsis, and respiratory failure (0.2 y)[9]
30	1) Systemic corticosteroids (PRED)[3]<br>2) HCQ[3]<br>3) HCQ/MTX/PRED[3]<br>4) Sirolimus<br>5) Systemic corticosteroids (MPS/DEX) + AZA	PD	MS	Bone, rectum, skin	Co	No	Toxicity (Grade III, pneumonitis, perimyocarditis)	PRED (tapered: 60 to 0 mg/day)	Alive (2.4 y)
31	VBL/PRED (LCH-A1 protocol)	PD	MS-RO-	Bone, cardiovascular system, kidney, lung, orbital cavity, pituitary, skin (external ear)	D + T	No	Treatment break (because of comorbidity-related fatigue)	No	Alive (11.7 y)
32	1) VBL/PRED<br>2) Bisphosphonates (APD; 1 infusion)	PD	MS-RO- (with ND lesions)	Bone, brain (ND lesions cerebellum and pons), kidney, skin	V	No	Death	No	Died from unknown cause (12 y)
33	1) ARA-C (6 cycles)<br>2) 2-CDA (2 cycles)<br>3) Radiotherapy (bone+mucosa, 26 Gy)	PD	MS-RO-	Bone, cardiovascular system, lymph node, oral mucosa (palate), pituitary	V	No	Death	MPS (1x 3 days), followed by PRED (20mg)	Died with progressive ND disease (7.2 y)

Case	Type of prior treatment(s)	Last response to prior treatment	Disease extent at targeted therapy	Sites of disease at start of targeted therapy	Inhibitor(s) at start	Targeted therapy ongoing (current dose)	Reason for stop of targeted therapy	Conventional treatment started during or after targeted therapy	Status (follow-up since diagnosis)
**Rosai-Dorfman disease**									
34	-[11]	-	MS	Lymph node, paranasal sinuses, skin, soft tissue (nasal septum)	Co		Not reportable		
35	1) Systemic corticosteroids (PRED)<br>2) Sirolimus<br>3) 2-CDA (6 courses)<br>4) MTX (max. 37.5 mg/week)	SD	MS	Bone, CNS (intraspinal meningeal mass at level S1-S3 extending into the neuroforamina and paraspinal muscles), kidney, lymph node, soft tissue (muscles; subcutaneous nodules)	T	Yes (once daily 0.5mg)	-	No	Alive (4.8 y)
36	1) Systemic corticosteroids (PRED/DEX)<br>2) 2-CDA/DEX (1 course)	PD	SS	Brain (multifocal tumors), spinal cord	Co	Yes (once daily 60mg, days 1-21/28)	-	No	Alive (4.0 y)
**Histiocytic sarcoma**									
37	Two ALL11 HR courses (without HD-MTX)[5]	PD	SS	Bone	T[8]	No	Received allogeneic HSCT	UCB HSCT with TBI, VP-16 and ATG as conditioning	Alive (1.4 y)
38	1) PRED + ICE (6 courses)<br>2) FLAG-IDA Salvage AML regimen (1 course)	PD	MS	Liver, lymph node, spleen	T		Not reportable		
**ALK-positive histiocytosis**									
39	Systemic corticosteroids (PRED)[3]	PD	SS	Soft tissue (lacrimal duct)	Cr		Not reportable		
**CNS-histiocytosis, unclassifiable**									
40	Systemic corticosteroids (MPS/DEX)	PD	SS	Brain (tumorous lesions periependymal region), peripheral nerves (trigeminal nerve), pituitary	V	Yes (twice daily 480mg)	-	No	Alive (2.0 y)

New abbreviations: VBL, vinblastine; MTX, methotrexate; 6-MP, mercaptopurine; PRED, prednisone; ARA-C, cytarabine; 2-CDA, cladribine; IC, induction course 1; DEX, dexamethasone; VP-16, etoposide; MPS, methylprednisolone; IVIG, intravenous immunoglobulin; VCR, vincristine; AZA, azathioprine; PEG-IFN-α, pegylated interferon-α; IFN-α, interferon-α; APD, pamidronate; HCQ, hydroxychloroquine; HR, high risk; HD, high dose; ICE, ifosfamide carboplatin etoposide regimen; FLAG-IDA, fludarabine cytarabine idarubicin and granulocyte colony-stimulating factor regimen; AML, acute myeloid leukemia; CNS, central nervous system; Gr., group; UCB, umbilical cord blood; HSCT, hematopoietic stem cell transplantation; TBI, total body irradiation; ATG, anti-thymocyte globulin.

Footnotes: [1] This is patient 4 from the study by Renier et al. (PMID 37250013). [2] The patient did not meet LCH-IV Stratum III criteria. The patient received a reduced dose of 2-CDA. [3] These treatments were given before the diagnosis of a histiocytic neoplasm was made. [4] This patient also received sarilumab (<2 months) as part of a clinical trial. [5] This does not include the prior treatments for the patient's B-ALL, before the diagnosis of secondary histiocytic sarcoma. For the initial B-ALL, this patient received chemotherapy (ALLTogether NCI HR protocol), followed by immunotherapy (2x CD19-directed CAR-T cell infusions; later 2 courses of 4 weeks of blinatumomab). The patient never achieved MRD negativity by PCR analysis of immunoglobulin gene rearrangements; the lowest MRD value was 0.02% at 6 weeks after first CAR-T cell infusion. [6] In addition to neurodegenerative lesions, this patient potentially had an orbital lesion. The orbital lesion was surgically excised; histopathologic analysis could not confirm LCH involvement. [7] This patient potentially had residual skin lesions. All other lesions present at diagnosis had resolved at time of onset of ND-LCH and subsequent start of targeted therapy. [8] Dasatinib was also given because of high *PDGFRB* expression in the B-ALL, according to RNA-sequencing data. [9] Death was presumably related to complications of an arterial bleeding of the duodenal bulb. During gastroscopy, two ulcers were seen in the duodenum (Forrest Classification: 1B), both with blood oozing. These were coagulated or clipped. The ulcers might have been prednisone-induced (the patient still received 60mg prednisone/day at start of targeted therapy), or the GI bleeding could have been cobimetinib-related. This is uncertain. Subsequently, the patient had 2 rebleeds, requiring coiling or embolization. Radiologic imaging revealed a large hematoma near the duodenum, as well as increasing pleural effusion and ascites – probably related to the severe LCH-related liver failure. The patient eventually died from sepsis (possibly caused by infected ascites/duodenal hematoma) and respiratory failure (caused by significant pleural effusion). [10] The patient received reduced doses (60%) of 2-CDA. [11] Prior to targeted therapy, this patient received antalgic radiotherapy (30 Gy in 10 fractions) of a lesion of the left elbow. Since the other lesions were not treated, radiotherapy was not considered as the 1st line of therapy.

SUPPLEMENTARY TABLE 2. Adverse events according to the Common Terminology Criteria for Adverse Events version 5.0

Case	Gr1	Gr2	Gr3	Gr4	Gr5	Inhibitor(s)	Description	Comments
1	**Yes**	-	-	-	-	V	Skin papilloma, palmar-plantar erythrodysesthesia syndrome, acneiform rash	
2	-	-	**Yes**	-	-	D + T	Gastrointestinal hemorrhage	Attributed to trametinib
3	-	-	**Yes**	-	-	V	Maculo-papular rash (painful; on torso and arms; doxycycline therapy started)	
4	-	-	**Yes**	-	-	T	Oral mucositis	In addition, unrelated grade 2 PJP and invasive aspergillosis infection
5	**Yes**	-	-	-	-	D + T	Weight gain	Attributed to trametinib
6	-	-	-	-	-	-		
7	-	**Yes**	-	-	-	V	Acneiform rash, decreased platelet count, fatigue, weight loss	
8	-	-	-	-	-	-		
9	**Yes**	-	-	-	-	V	Flu like symptoms, arthralgia, fatigue	
10	-	-	-	-	-	-		
11	-	-	-	-	-	-		
12	**Yes**	-	-	-	-	D	Skin papilloma, fever, myalgia, arthralgia	Skin papillomas disappeared after switch to dual dabrafenib/trametinib
13	-	-	-	-	-	-		During treatment with trametinib, the patient had tubulo-interstitial nefritis, which was attributed to other medication (deferasirox).
14	**Yes**	**Yes**	-	-	-	V	Grade 1: Myalgia, arthralgia, fatigue, alopecia.<br>Grade 2: Photosensitivy.	
15	**Yes**	-	-	-	-	D	Hyperkeratosis	Hyperkeratosis disappeared after switch to dual dabrafenib/trametinib
16	**Yes**	-	**Yes**	-	-	Grade 1: T<br>Grade 3: D	Grade 1: acneiform rash.<br>Grade 3: uveitis.	
17	**Yes**	-	-	-	-	D + T	Fever	
18	**Yes**	-	-	-	-	D + T	Fever, diarrhea	
19	-	**Yes**	-	-	-	T	Acneiform rash	
20	Not reportable							
21	**Yes**	-	-	-	-	D	Hyperkeratosis	Hyperkeratosis disappeared after switch to dual dabrafenib/trametinib
22	**Yes**	-	-	-	-	D	Myalgia, arthralgia	
23	**Yes**	-	-	-	-	D	Hyperkeratosis	Hyperkeratosis disappeared after switch to trametinib
24	Not reportable							
25	**Yes**	-	**Yes**	-	-	Grade 1: D<br>Grade 3: D + T	Grade 1: hyperkeratosis.<br>Grade 3: acute kidney injury	Also had grade 3 anemia and acute kidney injury during combination of trametinib with dasatinib; subsequently, trametinib was stopped.
26	-	-	-	-	-	-		
27	-	-	-	-	-	-		
28	-	**Yes**	-	-	-	V	Acneiform rash, dry skin, skin papilloma, eczema	
29	-	-	-	-	**Yes**	Co	Duodenal hemorrhage	The hemorrhage might have been related to treatment with prednisone (the patient still received 60mg prednisone/day at start of targeted therapy) and/or cobimetinib.
30	-	-	**Yes**	-	-	Co	Pneumonitis, pericarditis, myocarditis	
31	-	-	-	-	-	-		
32	**Yes**	-	**Yes**	-	-	V	Grade 1: fatigue.<br>Grade 3: photosensitivity.	
33	**Yes**	**Yes**	-	-	-	V	Grade 1: increased CPK and serum amylase.<br>Grade 2: increased lipase	
34	Not reportable							
35	**Yes**	-	-	-	-	T	Acneiform rash	
36	**Yes**	**Yes**	**Yes**	-	-	Co	Grade 1: hyperkeratosis, myalgia, edema limbs, increased CPK, blood LDH, and ALT.<br>Grade 2: arthralgia.<br>Grade 3: hypertension.	
37	-	-	-	-	-	-		
38	Not reportable							
39	Not reportable							
40	**Yes**	**Yes**	-	-	-	V	Grade 1: photosensitivity, skin papilloma, arthralgia, fatigue, diarrhea.<br>Grade 2: dysgeusia, alopecia.<br>Unclassifiable: erythema nodosum	
Total	**18**	**7**	**8**	**0**	**1**			
